# Thyroid Dysfunction as a Mediator of Organochlorine Neurotoxicity in Preschool Children

**DOI:** 10.1289/ehp.1003172

**Published:** 2011-06-30

**Authors:** Jordi Julvez, Frodi Debes, Pal Weihe, Anna L. Choi, Philippe Grandjean

**Affiliations:** 1 Harvard School of Public Health, Boston, Massachusetts, USA; 2 Faroese Hospital System, Tórshavn, Faroe Islands; 3 Institute of Public Health, University of Southern Denmark, Odense, Denmark

**Keywords:** longitudinal study, neurodevelopment, neurotoxicity syndromes, organochlorine compounds, prenatal exposure delayed effects, thyroid hormones

## Abstract

Background: Exposure to organochlorine compounds (OCs) can alter thyroid function in humans, and hypothyroidism during early life can adversely affect a child’s neurodevelopment.

Objectives: In this study we aimed to assess the relationship between developmental organochlorine exposures and thyroid function and the relationship between thyroid function and subsequent neurodevelopment.

Methods: A population-based birth cohort of 182 children was followed annually up to 5.5 years of age. The assessments included OC concentrations in maternal pregnancy serum and milk, clinical thyroid parameters in maternal and cord serum, and subsequent neuropsychological outcomes of the child, along with sociodemographic cofactors. Resin triiodothyronine uptake ratio (T3RU) was also assessed as an estimate of the amount of thyroxine-binding globulin (TBG) sites unsaturated by thyroxine. The T3RU is high in hyperthyroidism and low in hypothyroidism.

Results: The findings showed consistent inverse and monotonic associations between organochlorine exposure and T3RU after covariate adjustments. We observed no associations with other thyroid parameters. T3RU was positively associated with improved performance on most of the neuropsychological tests. For other thyroid parameters, the findings were less consistent.

Conclusions: The results suggest that OC exposures may decrease the T3RU during early life, which is a proxy measure of the binding capacity of TBG. In addition, minor decreases of the thyroid function may be inversely associated with a child’s neurodevelopment.

Organochlorine compounds (OCs) are environmental pollutants with lipophilic properties found in higher concentrations in animals at the top of the food web, including humans (Needham et al. 2005). The use of most persistent OCs has been banned, but they still remain in the environment, and the compounds accumulate in food webs (Chevrier et al. 2008; Needham et al. 2005).

Exposures to OCs such as certain polychlorinated biphenyl (PCB) congeners, dichlorodiphenyldichloroethylene (*p*,*p*´-DDE), dichlorodiphenyltrichloroethane (*p*,*p*´-DDT), and hexachlorobenzene (HCB) are associated with thyroid function disruption (Alvarez-Pedrerol et al. 2009; Chevrier et al. 2008; Jugan et al. 2010; Pearce and Braverman 2009) and central nervous system dysfunction (Korrick and Sagiv 2008; Morales et al. 2008). These environmental pollutants have been associated with reduced thyroid hormone (TH) levels in serum and adverse neuropsychological outcomes in experimental and epidemiologic studies (Chevrier et al. 2008; Korrick and Sagiv 2008; Morse et al. 1993; Pearce and Braverman 2009). Furthermore, a number of human studies have found an association between lower TH concentrations and adverse neuropsychological functioning in children and adults. These findings have public health significance because iodine deficiency leads to a reduction of THs (Alvarez-Pedrerol et al. 2007, 2009; Haddow et al. 1999; Pop et al. 1999; Rebagliato et al. 2010; Samuels 2008).

The complexity of the biological actions linking OC exposures to the disruption of the thyroid homoeostasis is not well understood, although there is evidence that interactions may take place at the receptor level or that the metabolism of THs may be affected. Similarities in the molecular structures of OCs, triiodothyronine (T_3_), and thyroxine (T_4_) may lead to competition for binding transport proteins, such as thyroxine-binding globulin (TBG) and transthyretin (TTR) (Alvarez-Pedrerol et al. 2009; Chevrier et al. 2008; Kirkegaard et al. 2010). Thus, in the presence of OCs, THs may be displaced from transport proteins and excreted at higher rates. Reduced TH may stimulate compensatory reactions, resulting in increased thyroid-stimulating hormone (TSH) levels (Kirkegaard et al. 2010).

THs are necessary for metabolism regulation and for maintaining normal cardiovascular, reproductive, and nervous system functions in humans (Samuels 2008). They also are essential for human fetal brain development, because they regulate dendritic processes, axonal growth, synaptogenesis, neuronal migration, and myelination (Morreale de Escobar et al. 2004). Research has shown that hypothyroidism during pregnancy and the perinatal period increases the risk of impairment to children’s long-term neuropsychological development (Haddow et al. 1999; Rovet and Hepworth 2001; Zoeller and Rovet 2004). In addition, a recent study found that increments of TSH and decrements of free T_4_ (FT_4_) concentration within normal ranges were adversely associated with neuropsychological outcomes in 4-year-olds (Alvarez-Pedrerol et al. 2007). Moreover, TTR and TBG transport T_4_ and other THs to the developing fetal brain and facilitate transfer of maternal TH across the placenta (Chevrier et al. 2008; Hume et al. 2004). TBG is the major T_4_ transport protein in human plasma, responsible for 75% of the T_4_ binding activity, but its physiological concentrations are also affected by estrogens, androgens, glucocorticoids, and drugs (Dallaire et al. 2008; Hume et al. 2004).

Thus, further studies examining the relationships between OC exposures and thyroid function during pregnancy and perinatal periods as well as subsequent child neurodevelopment in a population-based birth cohort are needed for several reasons. Lower TH levels even within normal ranges can adversely influence neuropsychological functioning (Alvarez-Pedrerol et al. 2007; Haddow et al. 1999; Henrichs et al. 2010). The human brain is particularly vulnerable to exposures that affect neurodevelopment during pregnancy and perinatal periods (Chevrier et al. 2008; Grandjean and Landrigan 2006), which possibly include environmental OCs that may act as endocrine disruptors and neurotoxicants (Pearce and Braverman 2009). It has been proposed that OC developmental neurotoxicity may result in part from OC-mediated impairment of thyroid function during the critical period of intense neurodevelopment (Chevrier et al. 2008), in addition to other mechanisms, for example, involving oxidative stress (Morales et al. 2008).

Some insights into the relationships among OCs, thyroid function, and neurodevelopment are based on experimental studies in laboratory animals and *in vitro* (Langer 2010; Morreale de Escobar et al. 2004). These studies have generally assessed the effects of OCs on T_3_, T_4_, and TSH levels, as well as thyroid transport proteins such as TTR (Langer 2010; Pearce and Braverman 2009). Human-based scientific literature in this area is scarce (Chevrier et al. 2008; Langer 2010; Morreale de Escobar et al. 2004; Pearce and Braverman 2009). To our knowledge, only one cohort study of 232 healthy mother–infant pairs has assessed OCs, thyroid function, and 2-year-old neurodevelopment together. The OC concentrations observed in this study were low, and no associations were identified (Wilhelm et al. 2008). Another cohort study reported that newborn TSH levels were inversely associated with cognitive development before and after adjusting for OC pesticide exposures measured in the placenta, although subjects were limited to 178 boys 4 years of age (Freire et al. 2010). Six other studies of OCs and thyroid function during pregnancy reported inverse associations between OCs and markers of thyroid function, particularly T_3_ concentration (Alvarez-Pedrerol et al. 2009; Chevrier et al. 2008; Koopman-Esseboom et al. 1994; Lopez-Espinosa et al. 2010; Steuerwald et al. 2000; Takser et al. 2005). In addition, several epidemiologic studies have examined associations between PCBs and TBG concentrations in human neonates, with contradictory results (Dallaire et al. 2008). Finally, some cohort studies have assessed prenatal exposures to OCs and neurodevelopment and have described adverse associations, but the role of thyroid function was not explored (Korrick and Sagiv 2008).

In the present study we aimed to ascertain whether environmental exposures to OCs are associated with thyroid parameters, including free triiodothyronine (FT_3_), FT_4_, total T_4_, free thyroxine index (FTi), resin triiodothyronine uptake ratio (T3RU), and TSH, all of which were measured during pregnancy and perinatal periods. Additionally, we explored associations between thyroid function and the child’s neuropsychological status assessed longitudinally up to 5.5 years of age.

## Materials and Methods

During a 12-month period in 1994–1995, a cohort of 182 singleton term births was generated from consecutive births at the National Hospital in Torshavn, Faroe Islands. Inclusion requirements included maternal residence in the central and northwestern region of the primary catchment area, that is, away from the capital area of Torshavn. About one-third of the Faroese population resides in this area, where OC exposure was presumed to have the greatest variation. About 64% of all eligible births were included. Four children who were born before the 36th week of gestation and two who had congenital neurologic disease were excluded. None of the children weighed < 2,500 g. Most of the relevant sociodemographic and obstetric data were obtained through in-person questionnaires and standardized procedures during pregnancy and after the children were born. There was annual follow-up, and the mothers completed the Home Observation and Measurement of the Environment (HOME) inventory (Bradley and Caldwell 1979) and the Raven IQ test when the children were 54 months of age (Steuerwald et al. 2000). The study design and methodology were approved by the Faroese Ethical Review Committee. Maternal serum was obtained during the last antenatal consultation at week 34. At delivery, the midwife drew blood from the umbilical cord by heparinized syringes with Teflon-lined pistons. Transition milk was obtained 4 or 5 days after parturition. All participating mothers gave written informed consent on each occasion.

*Neuropsychological measurements.* Neuropsychological tests were chosen based on other environmental studies of developmental neurotoxicity (Choi et al. 2008; Grandjean et al. 1997). The Bayley Scales (Bayley 1992) were used to assess each child’s general mental and psychomotor development at 30 and 42 months of age. Additionally, at ages 42, 54, and 66 months these scales were complemented with more specific tests to gauge brain function in the different domains. Details about test administration have been previously published (Choi et al. 2008; Grandjean et al. 1997). We included tests of visuospatial performance [Block Design test of Wechsler Preschool and Primary Scale of Intelligence (WPPSI) (Wechsler 1969), Block Design test of the Wechsler Intelligence Scale for Children–Revised (WISC-R) (Wechsler 1974), and Copying Block Design test (used in place of the Bender Test) (Choi et al. 2008)], language [Boston Naming Test (Kaplan et al. 1983)], and verbal fluency and short-term memory [California Verbal Learning Test (Children) (Delis et al. 1994)].

*Thyroid parameters.* TSH was measured in maternal serum and in cord serum by a time-resolved fluoroimmunoassay. FT_4_ and FT_3_ were measured by radioimmunoassay after dialysis to equilibrium. T3RU and total T_4_ were determined by radioimmunoassay, and FTi was calculated from T3RU and total T_4_ values (Steuerwald et al. 2000).

T3RU is an estimate of the amount of TBG sites unsaturated by T_4_. Radioactive T_3_ is used in the procedure, which is taken up either by the unsaturated TBG or by a resin binder. TBG has a greater affinity for T_4_ than for T_3_, so the radioactive T_3_ will not bind to TBG sites that are occupied by T_4_. Consequently, as T_4_ binding to TBG increases (e.g., in hyperthyroidism), more radioactive T_3_ will bind to the resin, resulting in a higher T3RU value. Conversely, as more T_3_ is taken up by TBG (e.g., when T_4_ levels are low), T_3_ uptake by the resin decreases and the T3RU will be lower (American Association of Clinical Endocrinologists 2002; Bakerman et al. 2002).

*Exposure biomarkers.* Serum organochlorine analysis. Two milliliters of all maternal serum samples were analyzed at the National Center for Environmental Health at the Centers for Disease Control and Prevention in Atlanta, Georgia (USA). Eighteen parent pesticides or their metabolites (i.e., *p*,*p*´-DDE and HCB) and 28 persistent PCB congeners were quantified by a two-stage solid-phase extraction method, followed by gas chromatography analysis with electron capture detection. The results were adjusted for total serum lipid content and reported as nanograms per gram lipid (Steuerwald et al. 2000). ΣPCBs was calculated as the sum of PCB congeners 138, 153, and 180, which we then multiplied by 2 [(PCBs 138 + 153 + 180) × 2] (Grandjean et al. 1995), to minimize the problems from concentrations of less common congeners with results below the limit of detection (LOD).

Milk organochlorine analysis. As an additional measure of perinatal exposure, 5 mL human milk was analyzed at the Institute of Environmental Toxicology in Kiel, Germany, for a similar array of OCs. After solid/liquid-phase extraction, analyses were performed by gas chromatography with electron capture detection (Steuerwald et al. 2000). ΣPCBs was again calculated as the sum of congeners 138, 153, and 180, which we then multiplied by 2 (Grandjean et al. 1995).

*Data analysis.* Measurements that deviated substantially from a Gaussian distribution, especially the OC exposure and thyroid parameters, were normalized by logarithmic transformation. Parametric methods were used whenever applicable. In the first part of the analyses, each log_10_-transformed thyroid parameter was modeled as a dependent variable, and individual biomarkers of organochlorine exposures were entered as log_10_-transformed continuous independent variables in separate regression models, with adjustment for covariates that were selected *a priori* and retained in the model if they predicted the outcome with *p*-values < 0.1. Additionally, an interaction term was included in the final models [smoking (yes/no) × log_10_-transformed ΣPCBs], aiming to assess the possible interaction between OC exposure and maternal smoking during pregnancy in relation to the thyroid parameters. Interactions with *p*-values < 0.10 were considered statistically significant. Trend tests (*p*-value for trend) were performed by transforming the OC exposures into quartile-categorical variables [first quartile (Q1) through fourth quartile (Q4)] and rerunning the corresponding regression models.

In the second part of the analyses, each neuropsychological function was modeled as a dependent variable, and each individual thyroid parameter was evaluated as a log_10_-transformed independent variable with adjustment for maternal serum ΣPCB concentration as a log_10_-transformed covariate. We used generalized additive models (GAMs) (Hastie and Tibshirani 1990) to assess linear trends with log_10_-transformed OCs as predictors and the generalized estimating equation (GEE) (Diggle et al. 1994) to model the repeated measurements of two sets of Bayley Scales (at 30 and 42 months) and three sets of copying block tests (at 42, 54, and 66 months). Regression coefficients were expressed as change in outcome (as a percentage of the standard deviation of the unadjusted outcome parameter) associated with a doubling of the independent parameter. This statistical technique was applied only when the outcomes were neuropsychological functions, because of the score range variability between the different tests. The α-level for statistical significance was 0.05 in all regression analyses.

## Results

Table 1 lists child and maternal anthropometric characteristics. Girls showed slightly lower weight at birth and 15 days after birth compared with boys; we found no sex-specific differences for other characteristics. Most of the mothers did not smoke or drink alcohol during pregnancy, and 93% breast-fed for > 1 month. Six mothers (3.3%) were < 18 years of age.

**Table 1:** Covariates of interest by children’s sex.

Covariate	Total (*n* = 182)	Boys (*n* = 93)	Girls (*n* = 89)
Child						
Gestational age (weeks)		40 (36–42)		40 (36–42)		39 (36–42)
Birth weight (g)		3,650 (2,500–4,800)		3,750 (2,700–4,800)		3,600 (2,500–4,500)
Birth length (cm)		53 (48–59)		53 (49–58)		52 (48–59)
Birth cranial circumference (cm)		35 (32–39)		36 (32–39)		35 (31–38)
Weight at 2 weeks of age (g)		3,980 (2,620–5,575)		4,095 (3,245–5,575)		3,800 (2,620–4,920)
Length at 2 weeks of age (cm)		55 (49–60)		55 (49–60)		54 (50–60)
Cranial circumference at 2 weeks of age (cm)		37 (33–40)		37 (35–39)		36 (33–40)
Exclusive breast milk (months)		4 (0–7)		4 (0–6)		4 (0–7)
Mother						
Age (years)		28 (16–44)		28 (16–43)		28 (17–44)
Parity before the child’s birth, > 1 [*n* (%)]		75 (41)		41 (44)		34 (38)
Weight before pregnancy (kg)		60 (45–109)		60 (45–109)		60 (45–93)
Height (cm)		163 (150–183)		163 (150–176)		162 (151–183)
Self-reported weight gain during pregnancy (kg)		14 (0–29)		15 (0–24)		13 (3–29)
Smoking during pregnancy, yes [*n* (%)]		57 (31)		26 (28)		31 (35)
Alcohol intake during pregnancy, yes [*n* (%)]		23 (13)		11 (12)		12 (13)
Level of education, college [*n* (%)]		39 (22)		19 (21)		20 (23)
Raven test score (median)		49 (15–59)		49 (35–59)		49 (15–57)
HOME inventory at 54 months of child’s age (*n* = 143)		43 (30–54)		43 (30–54)		44 (32–53)
Data are means and ranges, except as indicated.						

Table 2 shows the distribution of maternal and cord serum TH concentrations and child neuropsychological outcomes. We found no extreme values for any of these variables. The Pearson correlation coefficients between maternal and infant paired serum measures were moderate and statistically significant (*p* < 0.05) in most cases: FT_3_ (*r* = 0.31), T_4_ (0.27), T3RU (0.34), and FTi (0.20). The correlation coefficient for TSH (0.15) was also moderate (*p* < 0.10), and only FT_4_ (0.01) showed a null correlation coefficient. Correlation coefficients between T3RU and FT_4_ both in cord and in maternal serum were positive, moderate, and statistically significant (*r* = 0.35 and *r* = 0.49, respectively). Neuropsychological tests did not show any clear deviations from expectation. The longitudinal repeated measurements, such as the Bayley Scales and Copying Blocks, demonstrated consistent score increments as children aged.

**Table 2:** Thyroid parameters and neuropsychological outcomes in the Faroese birth cohort (*n* = 182).

Parameter	*n*	Mean	Range
Thyroid parameters (geometric means)						
Cord serum						
TSH (IU/L)		158		6.98		2.03–24.70
FT_4_ (pmol/L)		158		11.94		0.38–16.60
FT_3_ (pmol/L)		158		2.34		2.00–5.54
T_4_ (nmol/L)		158		128		68–205
T3RU		158		0.83		0.54–1.05
FTi (IU/L)		158		107		65–191
Maternal pregnancy serum						
TSH (IU/L)		173		1.34		0.23–3.61
FT_4_ (pmol/L)		174		8.17		5.49–14.70
FT_3_ (pmol/L)		174		4.25		2.94–6.62
T_4_ (nmol/L)		174		121		61–224
T3RU		174		0.66		0.51–1.01
FTi (IU/L)		174		80		53–164
Neuropsychological scores (arithmetic means)						
Bayley Scales at 30 months						
Mental		155		141		127–155
Motor		159		92		87–98
Tests at 42 months						
Bayley Mental Scales		154		158		141–171
Bayley Motor Scales		153		102		90–111
Verbal Fluency		154		12		7–15
Copying Blocks		139		4		1–9
Tests at 54 months						
Copying Blocks		164		6		2–12
WPPSI Block Design		160		9		0–18
Tests at 66 months						
Copying Blocks		169		8		1–12
WISC-R Block Design		168		13		4–19
Boston Naming		170		22		9–37

Table 3 describes the OC levels in the study population. ΣPCBs and *p*,*p*´-DDE showed higher concentrations than did HCB and *trans*-nonachlor. Paired maternal serum and milk OC concentrations showed high Pearson correlation coefficients: ΣPCBs = 0.90; *p*,*p*´-DDE = 0.93; HCB = 0.88; and *trans*-nonachlor = 0.68. Results for other OCs [*p*,*p*´-DDT, *p*,*p*´-DDD (dichlorodiphenyldichloroethane), β-HCH (β-hexachlorocyclohexane), aldrin, α-chlordane, dieldrin, γ-hexachlorocyclohexane, heptachlor, heptachlor epoxide, mirex, and oxychlordane] are not considered here because of a high proportion (> 60%) of results below the LOD.

**Table 3:** OC concentrations in the Faroese birth cohort (*n* = 182).

Concentration (μg/g)	
Pollutant	*n*	*n* < LOD	Geometric mean	Range
ΣPCBs				
Maternal serum	181	0	1.16	0.04–18.45
Milk	168	0	1.54	0.07–18.46
*p*,*p*´-DDE				
Maternal serum	181	0	0.72	0.20–8.04
Milk	168	0	0.88	0.05–13.71
HCB				
Maternal serum	181	0	0.08	0.03–0.66
Milk	168	0	0.06	0.01–0.41
*trans*-Nonachlor				
Maternal serum	181	24	0.04	0.00–2.06
Milk	168	6	0.09	0.00–1.67
PCBs (polychlorinated biphenyls); *p,p*´-DDE (dichlorodiphenyl dichloroethylene); HCB (hexachlorobenzene). ΣPCBs calculated from the sum of congeners 138, 153 and 180.				

Most OC concentrations presented in Table 4 showed crude and adjusted inverse associations with T3RU. We observed stronger associations for cord serum T3RU. FTi was inversely associated with the OCs, but the coefficients were not statistically significant after adjusting for covariates. Other thyroid parameters showed no associations in the multivariate models [Supplemental Material, Tables S1 and S2 (http://dx.doi.org/10.1289/ehp.1003172)]. Generally, associations with individual OCs were similar for OCs measured in maternal serum during pregnancy and breast milk after pregnancy. However, smoking during pregnancy was inversely associated with T3RU levels in this study (data not shown), and the T3RU geometric means in cord serum differed slightly between the groups (nonsmokers,0.84; smokers, 0.82); we observed no interactions between the exposure and smoking variables in the final models. Additionally, when we stratified the models by smoking, the OC coefficients were similar (data not shown).

**Table 4:** Crude and adjusted*a* β-coefficients (95% CIs) in thyroid parameters associated with a doubling of pollutant concentrations in regression analyses.^*b*^

T3RU							FTi (IU/L)						
Cord serum			Maternal serum			Cord serum			Maternal serum		
Pollutant	Crude	Adjusted*a*^,c^	Crude	Adjusted*a*^,c^	Crude	Adjusted*a*	Crude	Adjusted*a*
ΣPCBs																
Maternal serum		–0.011** (–0.021 to –0.002)		–0.012** (–0.022 to –0.001)		–0.006 (–0.015 to 0.002)		–0.007 (–0.019 to 0.005)		–1.5 (–3.8 to 0.8)		–0.9 (–2.8 to 1.0)		–1.4 (–3.1 to 0.2)		–2.5 (–5.5 to 0.6)
Milk		–0.010** (–0.019 to –0.001)		–0.010** (–0.021 to –0.000)		–0.004 (–0.014 to 0.005)		–0.005 (–0.020 to 0.010)		–1.5 (–4.0 to 0.8)		–0.7 (–2.8 to 1.3)		–0.6 (–2.5 to 1.3)		–0.8 (–4.3 to 2.8)
*p*,*p*´-DDE																
Maternal serum		–0.014** (–0.024 to –0.003)		–0.016^#^ (–0.028 to –0.004)		–0.006 (–0.016 to 0.004)		–0.012* (–0.025 to 0.001)		–1.6 (–4.1 to 1.0)		–0.5 (–2.8 to 1.7)		–2.3** (–3.9 to –0.7)		–2.8 (–6.3 to 0.7)
Milk		–0.011** (–0.019 to –0.002)		–0.014^#^ (–0.024 to –0.004)		–0.002 (–0.011 to 0.006)		–0.007 (–0.020 to 0.007)		–0.8 (–3.0 to 1.4)		–0.1 (–2.1 to 2.0)		–1.8** (–3.3 to –0.4)		–2.1 (–5.2 to 0.9)
HCB																
Maternal serum		–0.014 (–0.032 to 0.003)		–0.014 (–0.034 to 0.006)		–0.006 (–0.019 to 0.007)		–0.009 (–0.025 to 0.007)		–2.5 (–5.8 to 0.8)		–1.2 (–4.4 to 2.0)		–2.1** (–3.7 to –0.5)		–2.7 (–6.1 to 0.7)
Milk		–0.014** (–0.027 to –0.001)		–0.015* (–0.030 to 0.000)		–0.005 (–0.015 to 0.005)		–0.007 (–0.023 to 0.010)		–2.3* (–4.9 to 0.3)		–1.3 (–3.7 to 1.1)		–1.7** (–3.1 to –0.3)		–2.6 (–5.9 to 0.7)
*trans*-Nonachlor																
Maternal serum		–0.005^#^ (–0.010 to –0.001)		–0.006** (–0.010 to –0.001)		–0.004** (–0.007 to –0.001)		–0.005** (–0.010 to –0.001)		–0.4 (–1.4 to 0.7)		–0.1 (–1.0 to 0.8)		–0.5 (–1.2 to 0.1)		–1.0 (–2.2 to 0.3)
Milk		–0.013^#^ (–0.020 to –0.006)		–0.016^#^ (–0.023 to –0.008)		–0.003 (–0.009 to 0.003)		–0.006 (–0.014 to 0.003)		–1.6** (–3.2 to –0.1)		–1.0 (–2.4 to 0.3)		–1.0** (–2.1 to –0.0)		–1.3 (–3.3 to 0.5)
**a**Data adjusted for child’s birth weight and sex and maternal weight gain, age, and smoking during pregnancy. **b**Multivariate linear regressions were used after transforming the variables to fit a normal distribution and log transforming the exposure biomarkers. **c**The T3RU results were unchanged when the final models were adjusted by FT_3_ and FT_4_ as covariates (data not shown). *p < 0.10; **p < 0.05; ^#^*p* < 0.01.																

**Figure 1:**
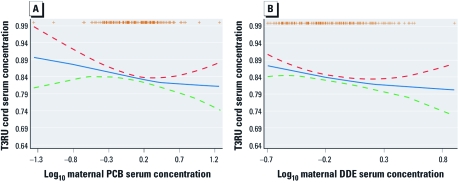
Associations between ΣPCBs (*A*) and *p*,*p*´-DDE (*B*) during pregnancy and T3RU in cord serum, adjusted for child’s birth weight and sex and maternal weight gain, age, and smoking during pregnancy (*n* = 151). The results were unchanged after adjusting for FT_3_ and FT_4_ as covariates (data not shown). Dashed lines indicate 95% CI for T3RU levels. Orange + signs at the top indicate observations.

The adjusted association between log_10_-transformed ΣPCBs and T3RU showed a linear trend pattern in GAMs (Figure 1A). We found similar results with log_10_-transformed *p*,*p*´-DDE and T3RU (Figure 1B). Associations with OCs modeled as categorical variables were consistent with those for OCs modeled as log_10_-transformed continuous variables. For example, coefficients from ΣPCB multivariate regression analyses using the lowest quartile (Q1) as the reference category were, for Q2, –0.02 [95% confidence interval (CI), –0.05 to –0.01]; Q3, –0.04 (–0.07 to –0.01); and Q4, –0.04 (–0.07 to –0.01); with *p*-trend = 0.017.

Although most associations between the OC concentrations and neuropsychological outcomes were weak, we observed an independent adjusted association between log_10_-transformed maternal serum ΣPCBs and Boston Naming scores at age 66 months [β = –5.23 (95% CI, –8.90 to –1.55)], as well as similar tendencies for *p*,*p*´-DDE [–5.14 (–8.81 to –1.49)], HCB [–10.87 (–17.46 to –4.28)], and *trans*-nonachlor [–1.49 (–2.99 to 0.00)]. We also noted inverse association tendencies that did not reach statistical significance for the other neuropsychological tests except for four outcomes (Block Design and Copying Block tests at ages 42 and 54 months). The ΣPCB associations with the Bayley Motor Scale at 30 months [–1.74 (–3.60 to 0.12)] and 42 months [–2.06 (–4.46 to 0.32)] were marginally significant. The ΣPCB coefficients were reduced by about 15% when we adjusted the same models with T3RU as a covariate: Boston Naming scores [–4.77 (–8.45 to –0.99)], 30-month Bayley Motor Scale [–1.50 (–3.46 to 0.46)] and 42-month Bayley Motor Scale [–1.72 (–4.24 to 0.78)].

Table 5 lists the adjusted associations between the thyroid parameters and the neuropsychological functions. TSH was inversely associated with block test at 66 months; FT_4_ was positively associated with Block Design tests at 54 and 66 months and with Copying Block test at 66 months; FTi was positively associated with Block Design test as well but inversely associated with Copying Block test at 66 months; and T_4_ was inversely associated with 54- and 66-month block tests and 30-month Bayley Motor Scale. Most of the thyroid parameters showed some inconsistencies with positive and reverse tendencies in the associations, particularly FTi, FT_4_, and TSH. T3RU showed the strongest positive associations with most of the neuropsychological functions assessed (Table 6). We observed only slight changes in the regression coefficients for T3RU after we included ΣPCBs as a covariate in the final models.

**Table 5:** Change in test score (expressed as percent of SD) associated with a doubling of the thyroid parameter in multiple regression analysis with adjustment for covariates.

TSH			FT_3_			FT_4_			FTi			T_4_		
Neuropsychological outcome	Cord	Maternal	Cord	Maternal	Cord	Maternal	Cord	Maternal	Cord	Maternal
Bayley Scales at 30 months																				
Mental		18.9*		–4.5		30.0		7.3		22.2		–43.0		47.4		–23.7		–24.0		–38.2
Motor		2.9		3.2		15.7		72.6*		16.4		35.9		–14.9		–40.8		–56.3		–62.4**
Tests at 42 months																				
Bayley Mental Scales		0.7		–1.6		–7.3		36.8		27.1*		–2.4		35.9		–18.5		–5.5		–44.9
Bayley Motor Scales		–9.7		–2.7		15.5		36.9		1.5		–6.7		32.0		–28.5		–25.2		–47.2
Verbal Fluency		13.9		18.0		24.9		–31.8		–4.1		–53.5		21.2		–45.9		–16.6		–43.9
Copying Blocks		–15.0		3.0		45.8		30.0		23.7*		48.6		7.0		1.2		–30.8		–45.8
Tests at 54 months																				
Copying Bocks		–17.1		1.7		–28.3		–5.1		28.3*		–1.5		4.8		17.7		–42.8		8.6
WPPSI Block Design		0.2		–11.1		–0.7		34.8		8.1		74.4**		–11.8		–10.0		–33.0		–61.1**
Tests at 66 months*a*																				
Copying Blocks		–16.6		–1.8		20.8		–26.0		32.7**		–40.6		56.2		–92.7^#^		8.2		–81.1^#^
WISC-R Block Design		–23.2**		–7.4		1.5		59.1		34.2**		29.7		80.1**		–2.8		32.8		–31.1
Boston Naming		–12.0		–11.0		14.2		29.5		13.2		27.6		22.4		13.4		–16.0		5.1
Each row shows results of one linear multivariate model adjusted for child’s sex, length at age 15 days, and chronological age at test administration and maternal parity, education, IQ (Raven test), and smoking during pregnancy. Multivariate linear regressions were used after transforming the variables to fit a normal distribution and log transforming the thyroid parameters. **a**Additional adjustment for testing location, language, having younger siblings, and day care. *p < 0.10; **p < 0.05; ^#^*p* < 0.01.																				

**Table 6:** Change in test score (expressed as percent of SD) associated with a doubling of T3RU in multiple regression analysis with adjustment for covariates and PCBs.

Cord serum			Maternal serum		
Neuropsychological outcome	Crude	Adjusted for ΣPCBs*a*	Crude	Adjusted for ΣPCBs*a*
Bayley Scales at 30 months								
Mental		189.1^#^		195.6^#^		53.5		53.5
Motor		134.4**		135.1**		84.2*		84.2*
Tests at 42 months								
Bayley Mental Scales		130.6**		140.1**		81.3*		82.9*
Bayley Motor Scales		183.1^#^		188.8^#^		69.0		69.4
Verbal Fluency		141.2**		132.3*		30.9		30.5
Copying Blocks		125.5**		143.7**		116.0**		116.8**
Tests at 54 months								
Copying Blocks		198.0^#^		218.2^#^		12.0		14.3
WPPSI Block Design		97.8		121.5*		137.8^#^		141.8^#^
Tests at 66 months*b*								
Copying Blocks		150.6**		151.5**		50.0		50.1
WISC-R Block Design		133.8**		133.8**		76.6*		76.6*
Boston Naming		130.7**		122.5*		12.7		12.4
Each row shows results of one linear multivariate model adjusted for child’s sex, length at age 15 days, and chronological age at test administration and maternal parity, education level, IQ (Raven test), and smoking during pregnancy. Multivariate linear regressions were used after transforming the variables to fit a normal distribution and log transforming the T3RU. The results were unchanged after adjusting for FT_3_ and FT_4_ as covariates (data not shown). **a**Adjusted for maternal serum ΣPCB concentration (μg/g lipid) as a log_10_-transformed covariate. The results were similar when including the maternal serum sum of 28 persistent PCB congeners as a log_10_-transformed covariate (data not shown). **b**Additional adjustment for testing location, language, having younger siblings, and day care. *p < 0.10; **p < 0.05; ^#^*p* < 0.01.								

The results of Tables 5 and 6 are comparable to results obtained in GEE models for the repeated measurements of Bayley Scales and copying block tests (data not shown).

## Discussion

The present study shows that environmental exposures to OCs, such as PCBs, *p*,*p*´-DDE, HCB, and *trans*-nonachlor, are inversely associated with T3RU during pregnancy and at birth. The findings were independent of the type of biological sample used to measure OC concentrations, whether maternal serum at 34th week of gestation or breast milk 5 days after delivery. We found no significant associations for TSH and other TH measures after covariate adjustments. In addition, T3RU, especially in cord serum, was positively associated with most of the neuropsychological outcomes examined. Adjusted regression models suggested that decreased thyroid function may be associated with neurobehavioral deficits that are similar to those related to OC exposures, and PCBs in particular. Levels of TSH, total T_4_, FT_4_, and FTi showed weaker and less consistent associations with child neurodevelopment than did T3RU.

The original contribution from this study results from the assessment of a wide range of thyroid parameters, including T3RU as an indirect measure of TH binding (Steuerwald et al. 2000). The validity of this study is supported by the thyroid and OC assessments being based on multiple sets of samples, such as maternal serum (both parameters), cord serum (thyroid measures), and breast milk (OC concentrations). Such repeated measurements are less frequently found in the scientific literature (Alvarez-Pedrerol et al. 2009; Chevrier et al. 2008; Pearce and Braverman 2009).

Previous findings about OCs and human thyroid function are not consistent, with some publications showing null results (Dallaire et al. 2008; Wilhelm et al. 2008), although a reduction of TH levels with OC exposure has been more clearly observed in laboratory animal and *in vitro* experiments (Alvarez-Pedrerol et al. 2009; Chevrier et al. 2008; Morse et al. 1993; Pearce and Braverman 2009). A major conundrum concerning these observational studies is the heterogeneity of subject samples in relation to sex (i.e., male children, pregnant women, and male workers), age (newborns, infants, children, youngsters, and adults), and sample size (some of them small) (Alvarez-Pedrerol et al. 2008a, 2008b, 2009; Chevrier et al. 2008; Freire et al. 2020; Hagmar et al. 2001; Koopman-Esseboom et al. 1994; Meeker et al. 2007; Steuerwald et al. 2000; Takser et al. 2005), thus reducing the comparability of the findings. Nevertheless, inverse associations between T_3_ levels and exposures to PCBs and HCB have been described in several studies among pregnant women, with weaker associations found between PCBs, HCB, and β-HCH and either FT_4_ or TSH (Alvarez-Pedrerol et al. 2009; Chevrier et al. 2008; Koopman-Esseboom et al. 1994; Lopez-Espinosa et al. 2010; Steuerwald et al. 2000; Takser et al. 2005). Inverse associations between perinatal exposure to persistent organic pollutants (including PCBs and DDE) and TH levels, particularly T_4_ and T_3_, have been reported by some studies (Darnerud et al. 2010; Maervoet et al. 2007). Finally, two studies among men and children found concurrent (cross-sectional) inverse associations between T_3_ and OCs (PCBs, DDT, and HCB) (Alvarez-Pedrerol et al. 2008b; Meeker et al. 2007). To our knowledge, this is the first epidemiologic study to report adverse associations between the environmental exposure to *trans*-nonachlor pesticide and T3RU; only a small study (*n* = 38) aimed to assess the associations between OC levels and thyroid parameters but did not report significant results in relation to *trans*-nonachlor as an individual biomarker (Bloom et al. 2009). The present findings support the hypothesis that OCs such as PCBs, *p*,*p*´-DDE, HCB, and *trans*-nonachlor may affect some thyroid parameters, as indicated by the decrements of T3RU levels. At the same time, the TH levels did not seem to be affected, and a direct inverse association between OC exposures and TH function was not demonstrated by this study, in terms of T_3_, T_4_, and TSH. Thus, T3RU may act as an indirect but sensitive marker of thyroid function.

Epidemiologic studies, unlike experimental studies, are not able to disentangle specific mechanisms. However, they can suggest some causal pathways (Alvarez-Pedrerol et al. 2009). Our results suggest some pathways involving the biological interactions among T_3_, T_4_, and TH transport proteins. The T3RU measurement helps estimate the availability of TBG, the protein that carries most of the T_3_ and T_4_ in the blood. The higher the level of unsaturated TBG, the lower the value of T3RU. Thus, T3RU values depend on both the levels of THs and TBG. Normally T3RU is high in hyperthyroidism and low in hypothyroidism in clinical subject samples, and it may be influenced by other factors, such as estrogen levels, hepatic function, and concurrent illness. Additionally, T3RU also depends on the presence of substances that may compete with THs at their binding sites. The TBG levels are lower in cord serum, accounting for the higher T3RU (American Association of Clinical Endocrinologists 2002; Bakerman et al. 2002). Our data are in agreement with expectations, including the FT_4_ correlation with T3RU and the higher cord serum T3RU compared with maternal pregnancy serum.

Several hypotheses exist as to the mechanisms that determine how OCs might mimic or decrease the biological action of THs (Chevrier et al. 2008; Pearce and Braverman 2009). One of the hypotheses suggests a direct link between OCs and the TH receptors localized within the hypothalamic–pituitary–thyroid axis, thereby interfering with TH impacts on the expression of genes sensitive to THs (Bogazzi et al. 2003; Chevrier et al. 2008; Meeker et al. 2007; Pearce and Braverman 2009). Another possible pathway is through an increase in the clearance of TH through the induction of thyroid-metabolizing enzymes. For example, a reduction of T_3_ levels may be explained by an inhibition of type I monodeiodinase, which converts T_4_ in peripheral sites to biologically active T_3_, or an activation of type III monodeiodinase, which in turn catalyzes the deiodination of T_4_ to reverse T_3_ and of T_3_ to 3,3´-diiodothyronine (Alvarez-Pedrerol et al. 2009; Meeker et al. 2007). It is also plausible that OCs, because of their structural similarities with THs, compete for transport protein binding sites in blood such as TBG and TTR (Alvarez-Pedrerol et al. 2009; Chevrier et al. 2008; Kirkegaard et al. 2010; Pearce and Braverman 2009). Given the observed data, it is not possible to disentangle whether OCs interact with thyroid binding proteins such as TBG at lower levels of T3RU when the T_4_/T_3_ binding capacity of serum proteins increases. Nevertheless, T3RU was the parameter most sensitive to OC exposures, and the coefficients were unchanged after adjusting for FT_3_ and FT_4_ concentrations in the final models.

The OC concentrations reported in the present study, particularly PCBs (geometric mean, 1.16 μg/g) and *p*,*p*´-DDE (0.72 μg/g) are among the highest reported, although similar DDE levels were reported in the Menorca (Ribas-Fitó et al. 2006) and California (Chevrier et al. 2008) cohort studies. In relation to HCB (geometric mean = 0.08 μg/g), the levels in the present study are similar to those in other studies (Alvarez-Pedrerol et al. 2009; Chevrier et al. 2008; Dallaire et al. 2008; Darnerud et al. 2010; Hagmar et al. 2001; Koopman-Esseboom et al. 1994; Lopez-Espinosa et al. 2010; Maervoet et al. 2007; Morales et al. 2008; Takser et al. 2005; Wilhelm et al. 2008).

THs are crucial for human neurobehavioral development (Alvarez-Pedrerol et al. 2007; Morreale de Escobar et al. 2004; Samuels 2008), but there are few published reports of populations with “normal” TH levels during pregnancy and at parturition and longitudinal neuropsychological assessments (Wilhelm et al. 2008). We designed the present population-based birth cohort with these specific parameters in mind, in an area of apparently sufficient iodine intakes due to the high frequency of seafood consumption (Steuerwald et al. 2000). The results revealed monotonic and consistent associations between T3RU and some TH levels (i.e., FT_4_ in cord serum) and child neurodevelopment. These findings, in consonance with those of other reports (Freire et al. 2010; Haddow et al. 1999; Henrichs et al. 2010; Pop et al. 1999; Rovet and Hepworth 2001), support the hypothesis that slight decrements in the TH levels during pregnancy and early life can adversely affect subsequent neuropsychological outcomes, perhaps with long-term consequences. The results also show some unexpected associations, particularly associations of higher maternal levels of total T_4_ and FT_4_ with lower performances in some tests. This finding may have been attributable to bias or random error, but a similar result was observed in another study reporting an inverse association between neonatal total T_4_ and subsequent neurodevelopment (Oken et al. 2009). More important, as described above, environmental OC exposures were inversely associated with T3RU. Thus, T3RU may reflect a joint effect of OCs, and their neurotoxic effects, to the extent that it is mediated via induction of thyroid dysfunction, which may be better expressed by the T3RU than by the chemical concentrations of the individual substances. This longitudinal study has several methodological strengths, including repeated assessments of OC, thyroid, and neurodevelopment data; a wide range of OC and thyroid parameters; and comprehensive information about neurodevelopment, health, nutrition, maternal IQ, home environment, and other sociodemographic characteristics.

## Conclusion

The present findings suggest that environmental exposures to PCBs and related substances diminish T3RU in pregnant women and newborns, although it does not seem to interfere directly with the concurrent TH levels. Furthermore, slight changes in T3RU and some thyroid parameters are associated with child neurodevelopment during subsequent years. Future epidemiologic studies should include assessments of the synergies among endocrine disruptors, thyroid function, and child neurodevelopment to help better understand these complex interactions and identify OC-mediated effects that may be particularly harmful in cases of decreased thyroid function, with plausible adverse consequences on future neurodevelopment.

## Supplemental Material

(156 KB) PDF
