# Supplementary material for: Thyroid Dysfunction as a Mediator of Organochlorine Neurotoxicity in Preschool Children
Source: Environ Health Perspect. 2011 Jun 30;119(10):1429–35. doi: 10.1289/ehp.1003172 (PMC3230434; doi:10.1289/ehp.1003172)
Supplement: (156 KB) PDF [file ehp.1003172.s001.pdf]

Supplemental Material

Thyroid Dysfunction as a Mediator of Organochlorine Neurotoxicity in Preschool Children

Jordi Julvez [1], Frodi Debes[2], Pal Weihe[2], Anna L Choi [1], Philippe Grandjean [1,3]

[1] Harvard School of Public Health, Boston, MA, United States

[2] Faroese Hospital System, Tórshavn, Faroe Islands

[3]Institute of Public Health, University of Southern Denmark, Odense, Denmark

*Corresponding author:

Jordi Julvez, Department of Environmental Health, Harvard School of Public Health, Boston, MA 02215, United States. Tel +1-617-384-8907. Fax: +1-617-384-8997. Email: jjulvez@hsph.harvard.edu

Table S1. Crude and adjusted[a] coefficients in thyroid parameters[b] associated with a doubling of pollutant[c] concentrations (μg/g) in regression analyses[d]

**Pollutant Concentrations**[a]	**TSH (IU/L) (Cord)** Crude	**TSH (IU/L) (Cord)** Adjusted[a]	**FT3 (pmol/L) (Cord)** Crude	**FT3 (pmol/L) (Cord)** Adjusted[a]	**FT4 (pmol/L) (Cord)** Crude	**FT4 (pmol/L) (Cord)** Adjusted[a]	**T4 (pmol/L) (Cord)** Crude	**T4 (pmol/L) (Cord)** Adjusted[a]
	Beta (CI)	Beta (CI)	Beta (CI)	Beta (CI)	Beta (CI)	Beta (CI)	Beta (CI)	Beta (CI)
**ΣPCBs (μg/g)**[e]								
Maternal Serum	-0.21 (-0.71 to 0.29)	-0.23 (-1.28 to 0.82)	0.01 (-0.05 to 0.07)	0.01 (-0.05 to 0.08)	0.01 (-0.23 to 0.25)	-0.03 (-0.48 to 0.43)	-0.3 (-3.3 to 2.9)	0.1 (-2.0 to 2.3)
Milk	-0.13 (-0.69 to 0.42)	0.11 (-1.10 to 1.31)	0.02 (-0.05 to 0.08)	0.02 (-0.05 to 0.09)	0.02 (-0.23 to 0.27)	0.01 (-0.42 to 0.45)	-0.5 (-3.7 to 2.6)	0.1 (-2.0 to 2.4)
***p,p'*-DDE (μg/g)**								
Maternal Serum	-0.04 (-0.65 to 0.57)	0.10 (-1.34 to 1.53)	0.00 (-0.07 to 0.07)	0.00 (-0.07 to 0.08)	-0.05 (-0.34 to 0.23)	-0.07 (-0.62 to 0.47)	0.1 (-3.5 to 3.7)	1.0 (-1.7 to 3.7)
Milk	-0.01 (-0.53 to 0.51)	0.37 (-0.94 to 1.69)	0.01 (-0.05 to 0.07)	0.01 (-0.06 to 0.08)	0.04 (-0.19 to 0.27)	0.07 (-0.34 to 0.47)	0.6 (-2.4 to 3.5)	1.3 (-1.0 to 3.5)
**HCB (μg/g)**								
Maternal Serum	-0.39** (-0.74 to -0.05)	-0.54 (-1.70 to 0.61)	0.01 (-0.11 to 0.13)	0.02 (-0.12 to 0.16)	-0.12 (-0.63 to 0.38)	-0.29 (-1.74 to 1.16)	-1.3 (-6.6 to 4.0)	-0.1 (-4.1 to 4.0)
Milk	-0.21 (-0.73 to 0.30)	0.31 (-1.94 to 2.57)	0.02 (-0.08 to 0.12)	0.03 (-0.09 to 0.14)	-0.02 (-0.39 to 0.34)	-0.12 (-0.94 to 0.70)	-1.2 (-5.1 to 2.8)	-0.1 (-3.2 to 3.0)
**Trans-Nonachlor (μg/g)**								
Maternal Serum	-0.03 (-0.26 to 0.19)	-0.07 (-0.53 to 0.40)	0.00 (-0.02 to 0.03)	0.01 (-0.02 to 0.04)	0.01 (-0.09 to 0.12)	0.03 (-0.14 to 0.21)	0.4 (-1.0 to 1.8)	0.5 (-0.5 to 1.5)
Milk	-0.11 (-0.47 to 0.25)	0.00 (-0.87 to 0.87)	0.01 (-0.04 to 0.05)	0.01 (-0.04 to 0.07)	-0.14 (-0.34 to 0.07)	-0.31 (-0.98 to 0.37)	-0.2 (-2.5 to 2.1)	0.4 (-1.3 to 2.2)

a: Child's birth weight and sex, maternal weight gain, maternal age and smoking during pregnancy ; b: TSH (thyroid-stimulating hormone); FT3 (free triiodothyronine); FT4 (free thyroxine); T4 (total thyroxine); c: PCBs (polychlorinated biphenyls); *p,p'*-DDE (dichlorodiphenyl dichloroethylene); HCB (hexachlorobenzene). d: Multivariate linear regressions were used after transforming the variables to fit a normal distribution and log transforming the exposure biomarkers. e: PCBs calculated from the sum of congeners 138, 153 and 180. * $p < 0.10$; ** $p < 0.05$; *** $p < 0.01$.

Table S2. Crude and adjusted[a] coefficients in thyroid parameters[b] associated with a doubling of pollutant[c] concentrations (µg/g) in regression analyses[d]

**Pollutant Concentrations**[a]	**TSH (IU/L) (Maternal)** Crude	**TSH (IU/L) (Maternal)** Adjusted[a]	**FT3 (pmol/L) (Maternal)** Crude	**FT3 (pmol/L) (Maternal)** Adjusted[a]	**FT4 (pmol/L) (Maternal)** Crude	**FT4 (pmol/L) (Maternal)** Adjusted[a]	**T4 (pmol/L) (Maternal)** Crude	**T4 (pmol/L) (Maternal)** Adjusted[a]
	Beta (CI)	Beta (CI)	Beta (CI)	Beta (CI)	Beta (CI)	Beta (CI)	Beta (CI)	Beta (CI)
**ΣPCBs (µg/g)**[e]								
Maternal Serum	-0.07 (-0.15 to 0.01)	-0.01 (-0.13 to 0.10)	0.03 (-0.05 to 0.11)	0.02 (-0.10 to 0.13)	-0.10 (-0.25 to 0.06)	-0.06 (-0.42 to 0.30)	-1.8 (-5.1 to 1.5)	-1.9 (-5.5 to 1.7)
Milk	-0.08* (-0.17 to 0.01)	-0.04 (-0.15 to 0.08)	0.04 (-0.04 to 0.13)	0.02 (-0.09 to 0.14)	-0.03 (-0.20 to 0.15)	0.09 (-0.38 to 0.56)	-1.1 (-4.6 to 2.4)	-1.0 (-4.6 to 2.7)
***p,p'*-DDE (µg/g)**								
Maternal Serum	0.02 (-0.09 to 0.12)	0.05 (-0.09 to 0.20)	-0.00 (-0.09 to 0.09)	0.01 (-0.11 to 0.13)	-0.10 (-0.27 to 0.08)	-0.16 (-0.52 to 0.20)	-2.2 (-6.0 to 1.6)	-0.2 (-4.3 to 3.8)
Milk	-0.01 (-0.09 to 0.07)	0.02 (-0.09 to 0.13)	0.02 (-0.05 to 0.09)	0.02 (-0.08 to 0.13)	-0.02 (-0.18 to 0.14)	-0.02 (-0.40 to 0.36)	-2.4 (-5.5 to 0.7)	-1.1 (-4.4 to 2.2)
**HCB (µg/g)**								
Maternal Serum	-0.04 (-0.18 to 0.11)	0.08 (-0.19 to 0.35)	0.07 (-0.13 to 0.26)	0.09 (-0.21 to 0.39)	-0.13 (-0.34 to 0.08)	-0.13 (-0.65 to 0.39)	-3.1 (-9.3 to 3.1)	-1.3 (-8.0 to 5.4)
Milk	-0.06 (-0.15 to 0.04)	0.04 (-0.16 to 0.24)	0.06 (-0.09 to 0.20)	0.04 (-0.16 to 0.22)	-0.08 (-0.28 to 0.11)	-0.05 (-0.60 to 0.33)	-2.7 (-7.5 to 2.1)	-2.8 (-8.1 to 2.4)
**Trans-Nonachlor (µg/g)**								
Maternal Serum	-0.02 (-0.05 to 0.02)	0.00 (-0.05 to 0.05)	-0.00 (-0.04 to 0.03)	-0.01 (-0.05 to 0.02)	-0.06** (-0.12 to -0.00)	-0.09 (-0.21 to 0.03)	-0.0 (-1.5 to 1.4)	-0.1 (-1.4 to 1.6)
Milk	-0.03 (-0.09 to 0.03)	-0.01 (-0.09 to 0.07)	-0.00 (-0.06 to 0.05)	-0.01 (-0.08 to 0.05)	-0.07 (-0.18 to 0.03)	-0.08 (-0.33 to 0.17)	-1.3 (-3.7 to 1.2)	-0.6 (-3.1 to 2.0)

a: Child's birth weight and sex, maternal weight gain, maternal age and smoking during pregnancy ; b: TSH (thyroid-stimulating hormone); FT3 (free triiodothyronine); FT4 (free thyroxine); T4 (total thyroxine); c: PCBs (polychlorinated biphenyls); *p,p'*-DDE (dichlorodiphenyl dichloroethylene); HCB (hexachlorobenzene). d: Multivariate linear regressions were used after transforming the variables to fit a normal distribution and log transforming the exposure biomarkers. e: PCBs calculated from the sum of congeners 138, 153 and 180. * $p < 0.10$; ** $p < 0.05$; *** $p < 0.01$.
